# Squamous Cell Carcinoma in a Calyceal Diverticulum Detected by Percutaneous Nephroscopic Biopsy

**DOI:** 10.1155/2018/3508537

**Published:** 2018-07-24

**Authors:** Taku Mitome, Tadashi Tabei, Yukio Tsuura, Kazuki Kobayashi

**Affiliations:** ^1^Department of Urology, Yokosuka Kyosai Hospital, 1-16 Yonegahamadori, Yokosuka, Kanagawa, Japan; ^2^Department of Pathology, Yokosuka Kyousai Hospital, 1-16 Yonegahamadori, Yokosuka, Kanagawa, Japan

## Abstract

A 73-year-old woman was referred to our department with a complaint of asymptomatic gross hematuria. Dynamic computed tomography revealed a complicated (Bosniak type IIF) cyst in the upper pole of her right kidney, which was diagnosed as a calyceal diverticulum. The diagnosis was confirmed by ureteroscopy. The diverticulum was filled with a soft protein matrix that was difficult to completely remove from the inner surface of the calyceal diverticulum. Endoscopy combined with intrarenal surgery (ECIRS) was performed to completely remove the matrix. Percutaneous nephroscopy further revealed papillary lesions on the surface of the diverticulum, confirmed as squamous cell carcinoma on pathological assessment. A laparoscopic right radical nephroureterectomy was performed, with curative intent. Pathological assessment confirmed a high-grade squamous cell carcinoma with renal parenchymal invasion (pT3). Although carcinomas in a calyceal diverticulum are highly uncommon, when present, these tend to be high-grade neoplasms that deeply invade the parenchymal wall. As the effective management of these lesions is difficult, early-stage diagnosis is required for curative treatment. We report the case of squamous cell carcinoma in a calyceal diverticulum that was difficult to diagnose on preoperative computed tomography, urinal cytology examination, and ureteroscopy but was found during ECIRS.

## 1. Introduction

Carcinoma in a calyceal diverticulum is largely uncommon. Although in most previous cases, the diagnosis was established on preoperative imaging, in one of these cases, the lesion was identified during percutaneous nephrolithotripsy [1]. We report a case of squamous cell carcinoma in a calyceal diverticulum that we identified using percutaneous biopsy and pathological analysis. Preoperative findings on computed tomography (CT), urinal cytology examination, and ureteroscopy were negative.

## 2. Case Presentation

**Figure 1:**
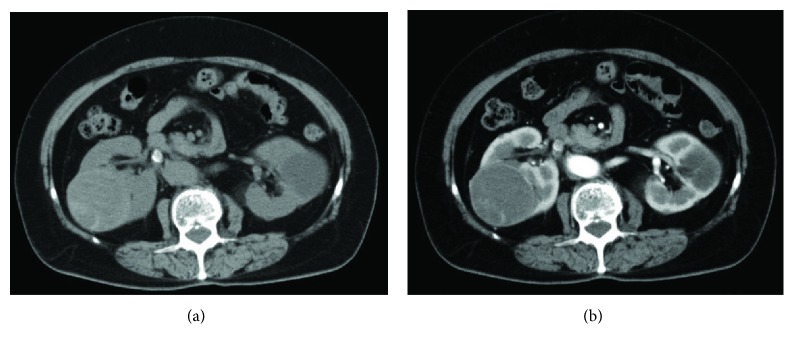
(a) Plain computed tomography image, showing a stone in the right upper urinary tract, but with no indication of the soft protein matrix in the right renal pelvis and calyceal diverticulum, which is radiolucent. (b) Dynamic computed tomography image, showing a complicated cyst, classified as a Bosniak type IIF cyst, arising from the right side of the kidney.

A 73-year-old woman was referred to our department with a complaint of asymptomatic gross hematuria. She had experienced a urinary tract infection and urolithiasis several years ago. On cystoscopy, we identified a gross hematuria from the right ureteral orifice. Noncontrast enhanced CT revealed a right renal stone and a complicated cyst in the upper pole of her right kidney, which was categorized as a Bosniak type IIF cyst on dynamic CT, with a maximum diameter of 58 mm (Figure 1).

**Figure 2:**
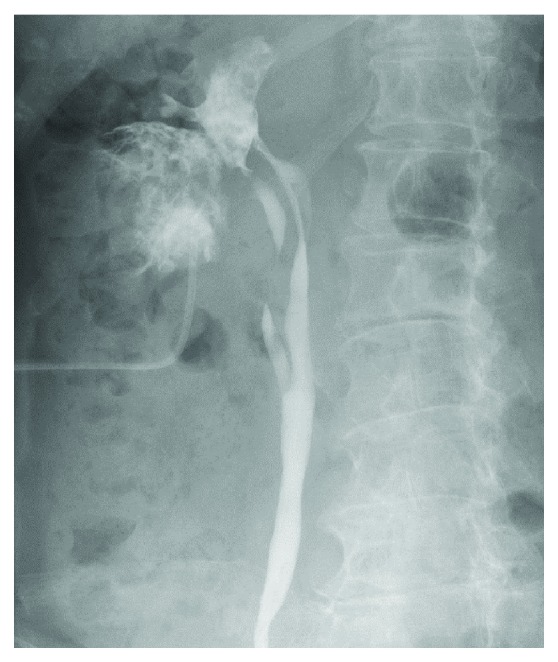
Retrograde pyelogram image, showing a duplex collecting system in the right kidney and calyceal diverticulum connected to the upper renal calyx. Defect sign of retrograde pyelogram, caused by filling of the diverticulum by the soft protein matrix, is observable. A percutaneous nephrostomy tube in the diverticulum was placed prior to ECIRS to remove the contents completely.

The renal stone was removed using flexible ureteroscopic lithotripsy. The calyceal diverticulum, which had been diagnosed preoperatively as a complicated cyst, was confirmed by ureteroscopy and retrograde pyelogram (Figure 2). As the diverticulum was filled with a soft protein matrix that was adherent to its wall, it was difficult to remove all the contents of the diverticulum while preserving the inner surface of the calyceal. With many fragments of the soft protein matrix floating in the calyceal diverticulum, renal pelvis, and ureter, obstruction of the ureter and ureteropelvic junction were predicted. Therefore, it was necessary to fully remove the matrix. Considering Bosniak type IIF classification of the cyst and the class II classification of the urine cytology examination, we proceeded with endoscopy combined with intrarenal surgery (ECIRS) to remove the contents completely, without follow-up observation.

**Figure 3:**
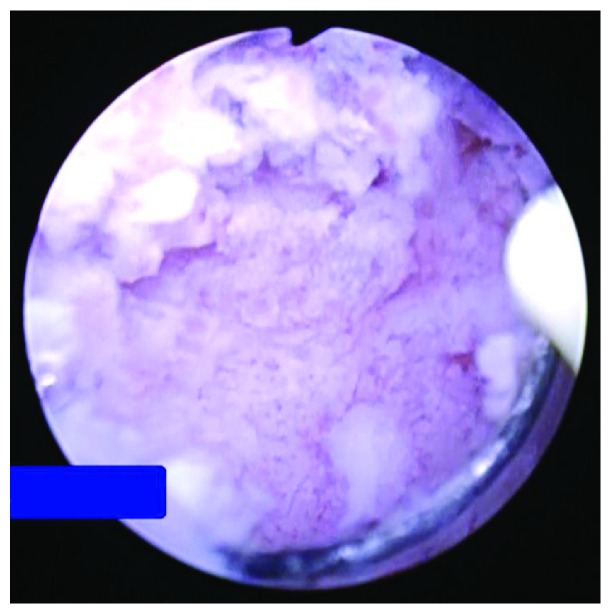
Percutaneous nephroscopic examination, showing papillary lesions in the calyceal diverticulum.

We punctured the diverticulum and dilated it using a 24 Fr balloon catheter (X-Force N30 Nephrostomy Balloon Dilation Catheter; Bard, New Providence, NJ, USA), under ultrasound guidance and ureteroscopy, with a working sheath placed at the edge of the diverticular cavity. After the soft protein matrix was completely removed, papillary lesions were observed on the surface of the diverticulum using percutaneous nephroscopy (Figure 3), and a biopsy was performed. Pathological analysis confirmed the diagnosis of squamous cell carcinoma, with the upper urinary tract stone composed of unspecified protein.

**Figure 4:**
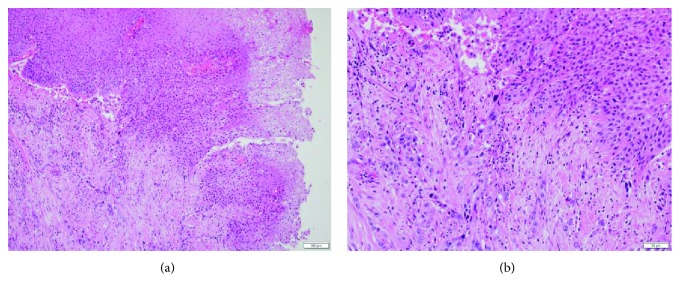
Histopathological examination. (a) An area of verrucous squamous cell carcinoma, connected to a high-grade squamous cell carcinoma, identified on the inner surface of the calyceal diverticulum. (b) Under magnification, the high-grade squamous cell carcinoma was shown to invade the renal parenchyma.

A right radical nephroureterectomy was performed using a laparoscopic approach, with curative intent. Pathological analysis confirmed a high-grade squamous cell carcinoma with renal parenchymal invasion (pT3) (Figure 4). Local recurrence was confirmed by CT imaging performed 2 months after that surgery, and the patient was treated with adjuvant systemic chemotherapy, using cisplatin and gemcitabine. The patient went into septic shock during the first course of chemotherapy, requiring cessation of systemic therapy, with her general condition worsening thereafter. At that point, the patient opted for palliative care only and she passed away 4 months after the radical nephroureterectomy was performed.

## 3. Discussion

Carcinoma in a calyceal diverticulum is extremely rare, with only 16 cases reported in the literature, to our knowledge. Moreover, only one case of primary squamous cell carcinoma in a calyceal diverticulum has previously been reported [1–7]. Among these 17 cases, including ours, the pathological findings included an urothelial carcinoma in 13 cases, urothelial carcinoma with squamous metaplasia in 2 cases, and squamous cell carcinoma in 2 cases [2]. In 11 of the 17 cases (64.7%), the tumor was classified as a high-grade carcinoma. In brief, the histological grade of carcinoma in calyceal diverticula tends to be high. Deep invasion in carcinoma into the wall of parenchyma is a notable clinical problem of these tumors, with a pT3 invasion of the carcinoma reported in 5 of the 17 cases (29.4%), including our case.

There is no consensus on the cause of calyceal diverticula. Dysfunction of the sphincters surrounding the calyces, which facilitate synchronized filling and emptying [3], has been proposed as one possible cause. Such calyceal achalasia results in chronic inefficient emptying, leading to progressive dilation proximal to the sphincter and subsequent formation of a diverticulum [3]. In this situation, the possible thinness or loss of the sphincter surrounding the mucosa of the calyceal diverticula may result in tumor invasion across the muscle layer of the sphincter [3]. Because of the risk of tumor invasion, any case of malignancy in a calyceal diverticulum should be carefully observed, with surgical treatment, with curative intent, performed as early as possible.

Calyceal diverticula often contain stones, and therefore, urolithiasis for stone removal should be performed in patients presenting with chronic pain, recurrent urinary tract infection, gross hematuria, or a decline in renal function [3]. Carcinoma in a calyceal diverticulum is also often associated with calculi, which were evident in 9 of the 17 cases reported (52.9%), including ours. Therefore, a possible diagnosis of malignancy should be considered in patients with calyceal diverticula. In cases of stone-related urothelial malignancy, chronic irritation and infection may play a significant role in the development of renal pelvis/ureter or bladder cancer [8].

In our case, pathological examination revealed a high-grade invasive squamous cell carcinoma, with squamous cell hyperplasia and verrucous carcinoma. There have been several studies on the correlation between squamous cell carcinoma and stones in the urinary tract [8]. Those pathological findings suggested that chronic irritation due to infection and the stones caused a transformation of the urothelium to squamous cell hyperplasia, accelerating the differentiation of squamous cell carcinoma.

In previously reported cases, the clinical diagnosis was exclusively based on preoperative imaging, with only one case identified during percutaneous nephrolithotripsy. Because in our case the diverticulum was classified as a Bosniak type IIF cyst and that the calyceal diverticulum was filled with a soft protein matrix, the diagnosis was difficult to make based on CT images and retrograde ureteroscopy. The decision to fully remove the soft protein matrix was made to prevent obstruction of the ureter and uteropelvic junction by the multiple fragments that were floating in the calyceal diverticulum, renal pelvis, and ureter. It was only after the matrix was completely removed, and the diverticular cavity was cleared that papillary lesions were observable, with a definitive diagnosis confirmed by percutaneous nephroscopic biopsy.

Naturally, seeding of carcinoma cells in the percutaneous tract during the percutaneous is of concern. However, a previous study reported a low incidence of seeding of urothelial carcinoma in the percutaneous tract, 0.3% overall and 0.75% (1/133) in the most experienced center [9]. Previous studies have reported the incidence of seeding of urothelial carcinoma in the percutaneous tract to be relatively low. Precautions to prevent seeding include the use of a large diameter sheath or low-pressure resection with continuous flow and avoiding the long-term implantation of a nephrostomy tube [10, 11]. Considering the low risk of seeding, we proceeded with percutaneous lithotripsy with all precautionary measures, although the presence of malignancy in the diverticulum could not be completely denied. As it is occasionally difficult to approach a calyceal diverticulum and observe its inner surface in detail, even with flexible ureteroscopy, we support the use of percutaneous nephroscopic biopsy as an option in patients with negative findings on preoperative CT and urinal cytology examination.

In summary, we report an extremely rare case of squamous cell carcinoma in a calyceal diverticulum that was diagnosed by percutaneous nephrosopic examination. The definitive diagnosis of carcinoma in a calyceal diverticulum can be difficult to confirm by imaging, urinal cytology examination, and ureteroscopy. In our case, the diagnosis was made during the percutaneous endoscopic procedure.
